# Comparative genomics reveals insights into genetic variability and molecular evolution among sugarcane yellow leaf virus populations

**DOI:** 10.1038/s41598-021-86472-z

**Published:** 2021-03-30

**Authors:** Jia-Ju Lu, Er-Qi He, Wen-Qing Bao, Jian-Sheng Chen, Sheng-Ren Sun, San-Ji Gao

**Affiliations:** 1 Guizhou Institute of Subtropical Crops, Guizhou Academy of Agricultural Sciences, Xingyi, 562400 Guizhou China; 2 National Engineering Research Center for Sugarcane, Fujian Agriculture and Forestry University, Fuzhou, 350002 Fujian China

**Keywords:** Microbiology, Plant sciences

## Abstract

Yellow leaf disease caused by sugarcane yellow leaf virus (SCYLV) is one of the most prevalent diseases worldwide. In this study, six near-complete genome sequences of SCYLV were determined to be 5775–5881 bp in length. Phylogenetic analysis revealed that the two SCYLV isolates from Réunion Island, France, and four from China were clustered into REU and CUB genotypes, respectively, based on 50 genomic sequences (this study = 6, GenBank = 44). Meanwhile, all 50 isolates were clustered into three phylogroups (G1–G3). Twelve significant recombinant events occurred in intra- and inter-phylogroups between geographical origins and host crops. Most recombinant hotspots were distributed in coat protein read-through protein (RTD), followed by ORF0 (P0) and ORF1 (P1). High genetic divergences of 12.4% for genomic sequences and 6.0–24.9% for individual genes were determined at nucleotide levels. The highest nucleotide diversity (π) was found in P0, followed by P1 and RdRP. In addition, purifying selection was a main factor restricting variability in SCYLV populations. Infrequent gene flow between Africa and the two subpopulations (Asia and America) were found, whereas frequent gene flow between Asia and America subpopulations was observed. Taken together, our findings facilitate understanding of genetic diversity and evolutionary dynamics of SCYLV.

## Introduction

Sugarcane yellow leaf virus (SCYLV) is a causal pathogen of yellow leaf disease (YLD) in sugarcane worldwide^1,2^. The first occurrence of YLD, previously known as yellow leaf syndrome, was described on sugarcane cultivar H 65-7052 in Hawaii in 1988^3,4^. Since then, the presences of SCYLV and sugarcane yellows phytoplasma (SCYP) were confirmed in plants during the 1990s and shared similar symptoms^5–8^. To discriminate the two diseases and their corresponding pathogens, Rott et al. proposed the disease caused by SCYLV was YLD, while leaf yellows was another disease resulting from SCYP^2^. Notably, a mix of SCYLV and SCYP infections has been found in sugarcane ^9–11^. Presently, SCYLV has been reported in more than 25 sugarcane growing countries, representing a major limitation to sugarcane production worldwide^12^.

SCYLV is phloem-limited and transmitted by various aphids in a semipersistent, circulative, and non-propagative manner, but not by mechanical transmission^13^. These aphids include *Ceratovacuna lanigera*, *Melanaphis sacchari*, *Rhopalosiphum rufiabdominalis*, and *R. maidis*^13–15^. Apart from aphid, long-distance transmission between sugarcane cultivation regions and countries is via SCYLV-infected cuttings^15–17^. SCYLV is limited to certain natural host plants, such as sugarcane (*Sachharum* spp. hybrids and other *Sachharum* species), species of *Erianthus*^13,14,18^, barley [*Hordeum vulgare*]^19^, grain sorghum [*Sorghum bicolor*]^20^, and Columbus grass [*Sorghum almum*]^21^. SCYLV infection has significantly affected the economic traits in sugarcane, such as cane growth, stalk diameter, number of millable canes, and sucrose content, which can lead to 10–50% loss in cane yield and 14% loss in sugar yield in plant or ratoon crops^12^. Moreover, about 30% yield reduction in asymptomatic sugarcane plants was reported in Thailand^22^.

In 2004, SCYLV was identified as a species of the *Polerovirus* genus of the *Luteoviridae* family^23^. Currently, 26 assigned species and some unassigned ones are included in the *Polerovirus* genus, which contains the representative species *Potato leafroll virus* (https://talk.ictvonline.org/taxonomy/). The genome of SCYLV is monopartite, 24–29 nm in diameter^14^, and consists of single-stranded, positive-sense, linear RNA (~ 6 kb) that include six open reading frames (ORFs 0–5) and three untranslated regions (UTRs)^24,25^. Additionally, two subgenomic RNAs (~ 2.4 and ~ 1.0 kb) in SCYLV found in either sugarcane^25,26^ or grain sorghum^27^ have been proposed during viral replication. Like other poleroviruses, translation of SCYLV proteins is carried out using a variety of strategies, such as leaky scanning, frameshifts, and read-through, to produce P1 (encoded by ORF1), RNA-dependent RNA polymerase (RdRP; P1–P2 fusion protein), and coat protein (CP) read-through protein (RTD), respectively^17^.

SCYLV has high genetic diversity among geographical origins and a dozen genotypes present worldwide which have been revealed by phylogenetic analysis based on complete genome sequences, including eight genotypes [BRA, CHN1, CHN3, CUB, HAW, IND, PER, and REU]^28^ and a few new genotypes, such as FLA1–FLA3^27,29^. Two more SCYLV genotypes (COL in Colombia and CHN2 in China) were proposed based on partial genome fragments^30,31^. Ancestors of SCYLV evolved through RNA recombination between species of three genera, *Luteovirus*, *Polerovirus*, and *Enamovirus*^24,25^. Subsequently, numerous studies have shown that some novel SCYLV isolates were regenerated by RNA recombination among worldwide isolates, and potential recombination hotspots were distributed in ORF1/2 and ORF5 regions^27,28,32^. Overall, viral RNA recombination is known to be an important evolutionary force in the SCYLV genome and represents a formidable challenge in managing YLD.

Breeding new resistant cultivars is critical for successful control of sugarcane diseases^33^. However, plant resistance is currently limited to one or some specific viral strains and for a short time (a few years) because plant virus is able to rapidly circumvent host acquired genetic resistance^17^. Usage of healthy seed cane is another conventional and effective management strategy for control of viral disease, including YLD^10,34^. The present study analyzed the genetic variability, molecular evolution, and population constructs of SCYLV isolates collected from Réunion Island, France, and China along with all published sequences in GenBank. The results of this investigation will enrich current knowledge of SCYLV genomes and understanding of the genetic diversity and evolutionary dynamics of this virus, especially at the global level, providing a vital basis for designing strategies and management schemes for YLD.

## Results

### SCYLV genome sequencing and assembly

Two nearly complete genomes of SCYLV isolates (REU-YL11 and REU-YL15) from Réunion Island were assembled based on six overlapping genomic fragments. Meanwhile, four nearly complete genomes of SCYLV isolates (ZJWL002, ZJWL003, ZJWL007, and ZJWL012) from Zhejiang Province, China, were assembled according to three overlapping genomic fragments. The six nearly full genome sequences were determined to be 5775–5881 bp in size (Table S1) and have been deposited in the National Coalition Building Institute (NCBI) under accession numbers KY052165 and KY052166, MW439312, MW439313, MW446950, and MW446951, respectively.

### Phylogenetic grouping of SCYLV isolates

**Figure 1:**
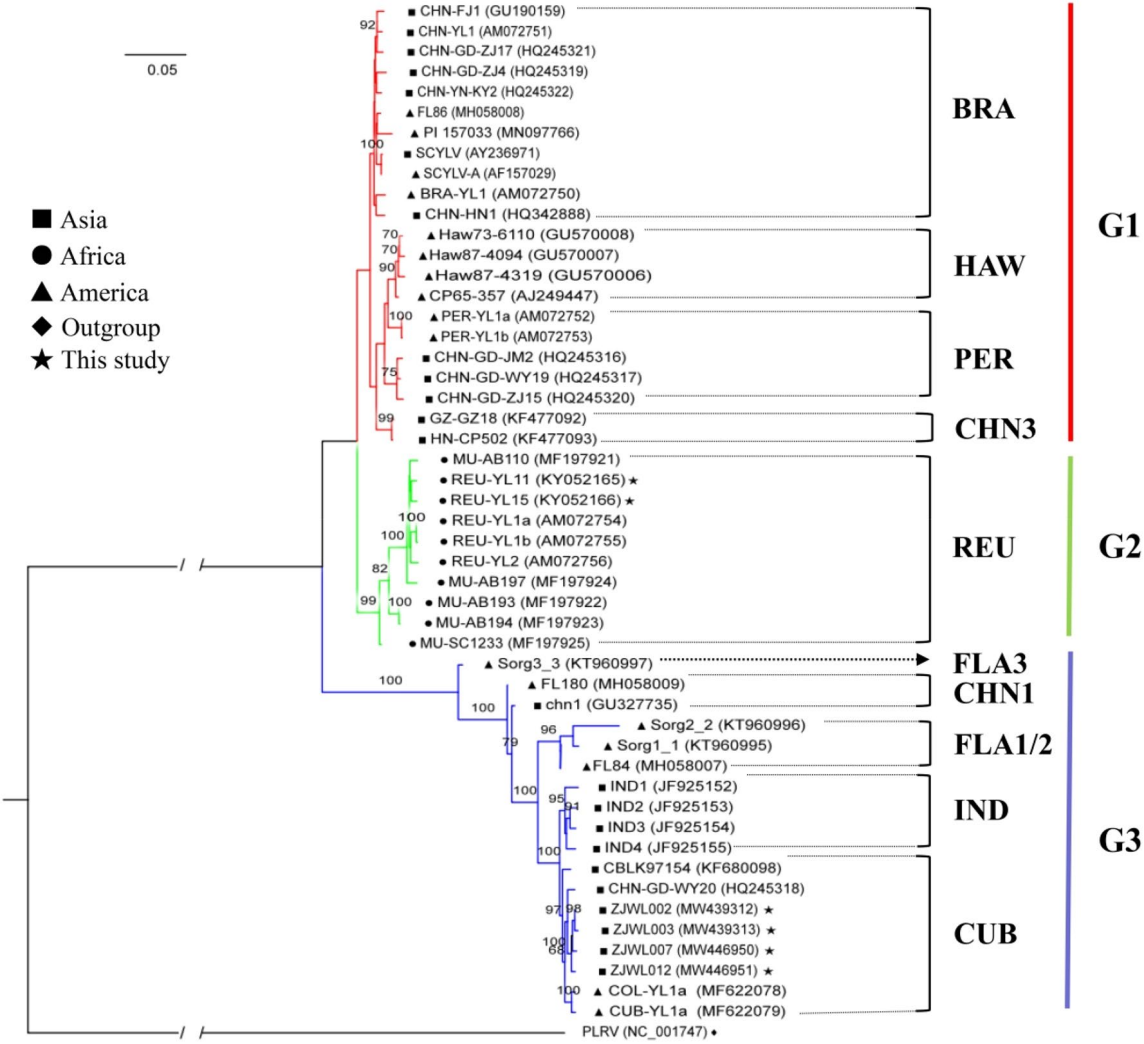
Maximum Likelihood (ML) phylogenetic tree constructed using IQ-TREE 1.6.12 based on nearly complete genome sequences [open reading frames (ORFs) 0–5] from 50 sugarcane yellow leaf virus (SCYLV) isolates. ML analysis was carried out using nucleotide identity distances and 1000 bootstrap replicates (Bootstrap values > 60% are shown at nodes). The scale bar indicates the number of substitutions per nucleotide. Different geographic regions are represented with red, green, and purple lines for Asia (filled square), Africa (filled circle), and America (filled triangle), respectively. The six SCYLV isolates from sugarcane obtained in the present study are indicated by the solid pentagram (filled star). A potato leafroll virus (PLRV; NCBI accession no. NC_001747) isolate served as an outgroup (filled diamond). The acronyms of each SCYLV genotype based on their originally geographical origins: BRA (Brazil), HAW (Hawaii), PER (Peru), CHN (China), REU (Reunion), FLA (Florida), IND (India), and CUB (Cuba).

The 5′-UTR and 3′-UTR sequences of all tested SCYLV genomes were trimmed as most isolates lacked these sequences. After that, the nucleotide sequences (ORFs 0–5) of 50 SCYLV isolates, including six sequences obtained in the present study, were subjected to phylogenetic analyses. The Maximum Likelihood (ML) phylogenetic tree revealed a clear segregation of all 50 isolates into three major clades (Fig. 1). Clade I (phylogroup 1, G1) consisted of four genotypes (BRA, CHN3, HAW, and PER), including 22 SCYLV isolates from Brazil, China, India, Peru, and USA. Interestingly, one isolate (PI 157033) in this group originated from sorghum (Sorghum bicolor). Clade II (phylogroup 2, G2) was comprised of a unique REU genotype, including 10 SCYLV isolates from Réunion Island and Mauritius, among which two isolates (REU-YL11 and REU-YL15) determined herein, were clustered in this genotype. Clade III (phylogroup 3, G3) contained the remaining 18 SCYLV isolates from China, Colombia, Cuba, India, and USA, including four isolates (ZJWL002, ZJWL003, ZJWL007, and ZJWL012) obtained in the current study that clustered in the CUB genotype. Apart from CHN1, CUB, and IND genotypes involved in phylogroup G3, two SCYLV isolates (Sorg1_1 and Sorg2_2) from grain sorghum along with one isolate (FL84) from sugarcane were assigned to genotype FLA1/2, while one unique isolate (Sorg3_3) from grain sorghum was proposed to be in a distinct group [FLA3] (Fig. 1).

### Detection of recombination signals

Recombination analysis revealed that 12 clear recombinants were observed among the 50 SCYLV isolates that were under the threshold of at least four of the seven recombination detection algorithms and had a high acceptable *P* < 0.05 (Table 1). These recombination isolates included GZ-GZ18 and PI 157033 from group G1, MU-AB193 and MU-SC1233 from group G2, and IND2 along with Sorg1_1, Sorg2_2, and Sorg3_3 from phylogroup G3. Seven extremely significant recombination signals were found among isolates that were under the threshold of seven algorithms with a high acceptable *P* < 0.05. All 12 significant recombination signals were further confirmed by the SimPlot program (Fig. S1). Apart from intra-phylogroups, such as GZ-GZ18 originating from CHN-GD-WY19 (major parent) and FL86 (minor parent), recombination events commonly occurred in inter-phylogroups, such as four isolates (PI 157033, Sorg1_1, Sorg2_2, and Sorg3_3) from grain sorghum clustered in group G3 which were generated from isolates between phylogroups G1 and G3 (Table 1). Meanwhile, recombination events commonly occurred within sugarcane and between sugarcane and grain sorghum. Additionally, while recombination events distributed across the whole genome, most recombination breakpoints (67%) presented at the 3′-terminal of the genome, followed by 25% recombination breakpoints at the 5′-terminal of genome, and only two recombination breakpoints were positioned in the middle region of the genome. These results indicated that SCYLV genomic regions of 3′- and 5′-termini are recombination hotspots.

**Table 1:** Recombination events among 50 sugarcane yellow leaf virus (SCYLV) isolates using RDP4 software.

Putative recombinant^a^	Major × minor putative parents ^a^	Recombination breakpoint^b^		Detection method^c^						
		Begin	End	R	G	B	M	C	S	T
PI 157033 (G1)	IND2 (G3) × BAR-YL1 (G1)	5001	5238	++	++	++	++	++	−	++
Sorg1_1 (G3)	FL86 (G1) × FL84 (G3)	624	728	++	++	++	++	++	−	++
MU-AB193 (G2)	CHN-GD-ZJ4 (G1) × MU-AB197 (G2)	2579	4506	++	++	++	++	++	++	++
Sorg3_3 (G3)	SCYLV (G1) × ZJWL003 (G3)	4503	5001	++	++	++	++	++	++	++
MU-SC1233 (G2)	CHN-YN-KY2 (G1) × MU-AB193 (G2)	4507*	5214	++	++	++	++	++	++	++
MU-AB193 (G2)	PI 157033 (G1) × REU-YL2 (G2)	1093	1463	++	++	++	++	++	++	++
MU-SC1233 (G2)	CHN-GD-ZJ15 (G1) × MU-AB197 (G2)	2556	4506*	++	+	−	+	+	++	++
GZ-GZ18 (G1)	CHN-GD-WY19 (G1) × FL86 (G1)	4437	5468	++	++	++	++	++	++	+
Sorg2_2 (G3)	FL86 (G1) × IND2 (G3)	4976	5178	++	+	++	+	++	++	++
Sorg3_3 (G3)	SCYLV (G1) × FL180 (G3)	387	521	++	++	−	++	++	−	+
Sorg1_1 (G3)	PI 157033 (G1) × Sorg3_3 (G3)	4257	4343*	++	+	+	−	+	−	+
IND2 (G3)	FL180 (G3) × CBLK97154 (G3)	5026	5237	++	++	++	+	+	+	+

^a^Phylogroups are shown in brackets.

^b^Actual breakpoint position undetermined (it was most likely overprinted by a subsequent one) shown as an asterisk (*).

^c^Seven algorithms implemented in RDP4 software were used, including RDP (R), GENECONV (G), BootScan (B), Maximum Chisquare (M), Chimaera (C), Sister Scan (S), and 3Seq (T). Symbols are showed for recombination signals at different statistical significance levels: ++, *P* ≤ 10^–6^; +, 10^–6^ < *P* ≤ 0.05; −, *P* > 0.05 (not significant).

### Sequence identity analysis within and between phylogroups

**Figure 2:**
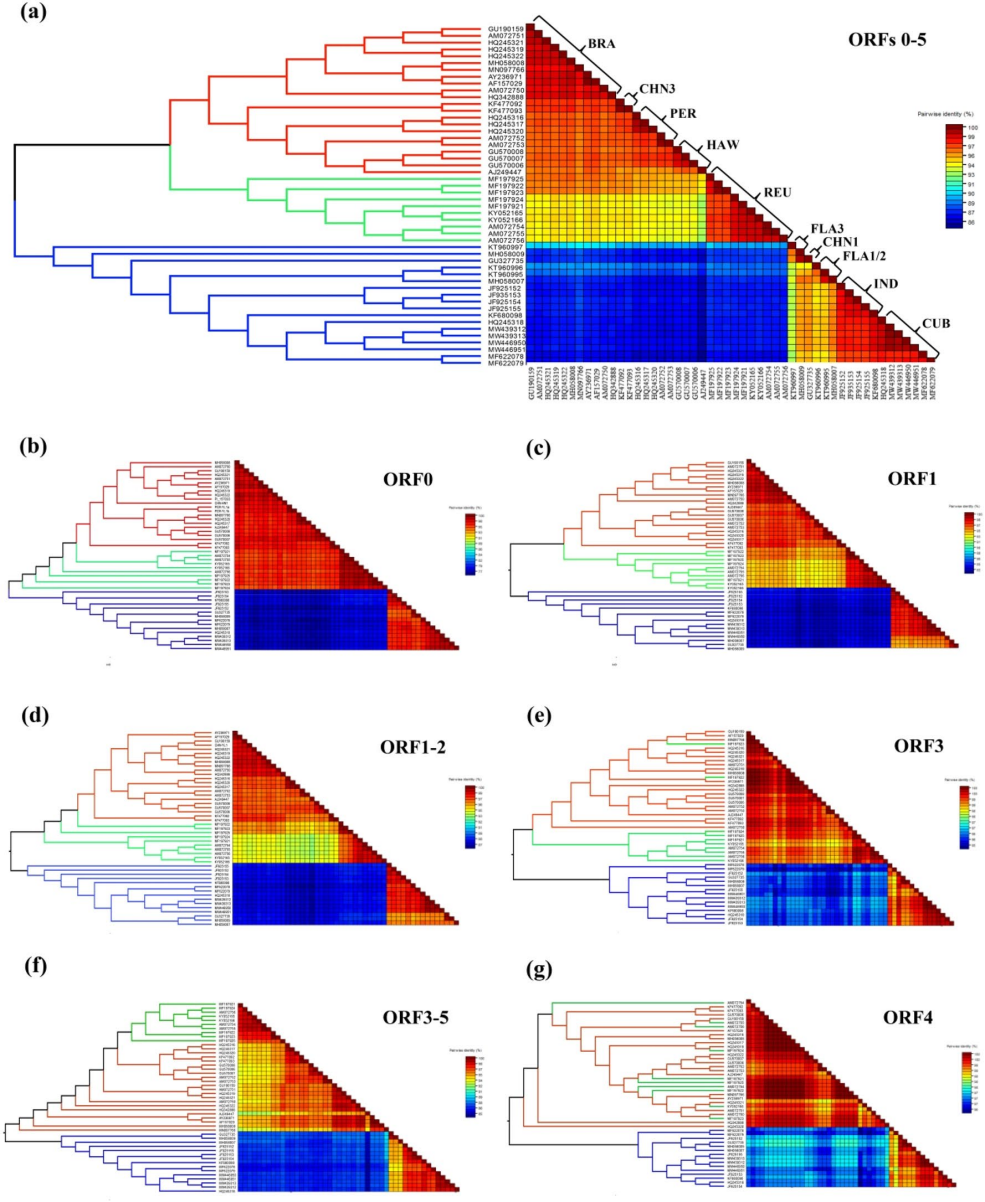
Maximum Likelihood phylogenetic trees (leaf panel) constructed by IQ-TREE v1.6.12 and pairwise identity matrixes (right panel) constructed by SDT v1.2 among SCYLV isolates. (**a**) Fifty nearly-complete genome sequences of SCYLV. (**b**)–(**g**) Six nucleotide sequences of ORF 0, ORF 1, ORF 1–2, ORF 3, ORF 4, and ORF 3–5 from 47 SCYLV isolates (Sorg1_1, Sorg2_2, and Sorg3_3 were excluded), respectively. The acronyms of each SCYLV genotype: BRA (Brazil), HAW (Hawaii), PER (Peru), CHN (China), REU (Reunion), FLA (Florida), IND (India), and CUB (Cuba).

Pairwise sequence identity analysis revealed that nucleotide sequence identities ranged from 83.7 to 99.9% among genome sequences (ORFs 0–5) from the 50 isolates, indicating high sequence diversity between isolates (Table S2). Obvious divergences between phylogroups were found, especially for the comparisons of phylogroup G3 with phylogroups G1 and G2 having more than 12.4% nucleotide variation (Fig. 2a and Table S2). After three SCYLV isolates (Sorg1_1, Sorg2_2, and Sorg3_3) were excluded from the analysis of genetic diversity, ML phylogenetic trees and sequence identities demonstrated significant variation between SCYLV phylogroups based on each gene and corresponding protein from 47 SCYLV isolates (Fig. 2b–g and Table S2). High divergence in nucleotide (nt) and amino acid (aa) sequences of SCYLV genes/proteins between phylogroups were found: 3.0–23.9% (nt) and 2.0–31.7% (aa) for ORF0 (P0), 3.7–20.1% (nt) and 4.0–27.9% (aa) for ORF1 (P1), and 3.4–15.4% (nt) and 3.0–29.9% (aa) for ORF1-2 (RdRP). By contrast, high sequence identities of ORF3 (CP) and ORF4 (MP) between phylogroups were ≥ 94.9% (ORF3) and ≥ 96.2% (ORF4) at nt levels and ≥ 93.3% (CP) and ≥ 95.3% (MP) at aa levels, respectively.

### Genetic variation in individual genes among phylogroups

**Figure 3:**
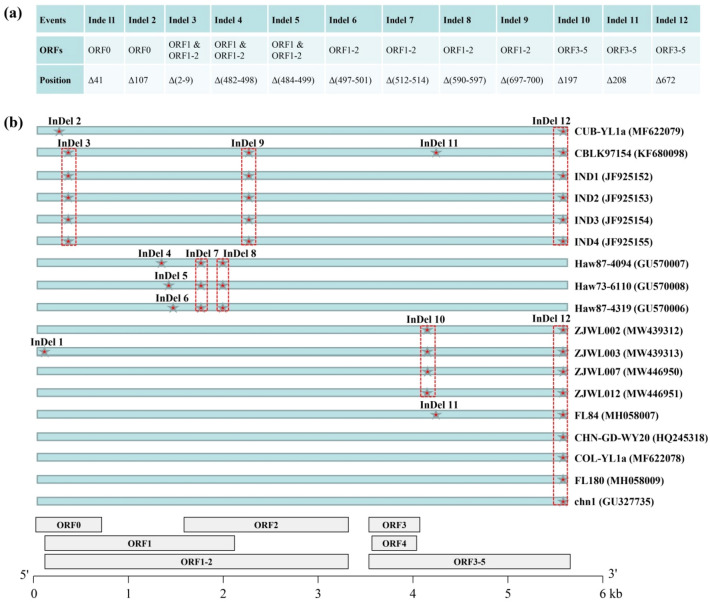
Schematic sketch of 12 Insertion/Deletion (InDel) events calculated by using amino acid sequences of 47 SCYLV isolates. (**a**) Position of InDel events in each ORF. (**b**) InDel events are indicated by the red pentagram (filled star), and the same InDel event occurring among SCYLV isolates is shown by the red dotted line. All isolate sequences (from ORF 0 to 5) are indicated by blue diagrams.

The 47 SCYLV isolates had complete ORFs and corresponding proteins, but slight differences in length in each ORF or protein (Table 2) were present due to 12 insertions/deletions (InDels) events occurring in 18 isolates (Fig. 3). The number of amino acids in these InDels ranged from one to 16 (Fig. 3a). These InDel events were distributed in different proteins: ORF0 encoding P0 (2 InDels), ORF1 encoding P1 (3 InDels), ORF1/2 encoding RdRP (4 InDels, pulsing 3 InDels in ORF1 region), and ORF3/5 encoding RTD (3 InDels). Notably, 15 of 18 isolates shared InDel 12, while the four SCYLV isolates hosted in a chewing cane clone Guangdong Huangpi (ZJWL002, ZJWL003, ZJWL007, and ZJWL012) from China specifically had InDel 10 (Fig. 3b). In addition, based on the sequence identities (nt and aa) and nucleotide diversity (π) in the overall SCYLV population (47 isolates was considered as a whole) or each phylogrouping subpopulation, higher genetic variation is present in the 5′-end of the viral genome, particularly in ORF0 (P0), and the lower genetic variation is present in the middle region of the viral genome, including ORF3 (CP) and ORF4 (MP) (Table 2). Among 47 SCYLV isolates, sequence identities of ORF0 were 91.7% and 88.6% at nt and aa levels, respectively, while nucleotide diversity (π) was 0.10735. By contract, ORF3 had 98.4% (nt) and 98.8% (aa) sequence identities, while nucleotide diversity (π) was 0.02242.

**Table 2:** The length, identity, nucleotide diversity, neutrality test, and selection pressure on each gene among the overall sugarcane yellow leaf virus (SCYLV) population and each phylogroup.

ORF (protein)	Group	Length		Identity (%)		π	Tajima's D	dN/dS
		aa	nt	aa	nt			
ORF0 (P0)	all (n = 47)	255–256	765–768	88.61	91.72	0.10735	1.48728 (ns)	0.4018
	G1 (n = 22)	256	768	97.37	98.43	0.02458	− 0.66815 (ns)	0.3564
	G2 (n = 10)	256	768	98.87	99.25	0.01195	− 0.04311 (ns)	0.2966
	G3 (n = 15)	255–256	765–768	91.15	98.06	0.02837	− 0.33086 (ns)	0.1467
ORF1 (P1)	all (n = 47)	633–650	1899–1950	91.26	92.96	0.09007	1.17081 (ns)	0.2774
	G1 (n = 22)	633–650	1899–1950	97.67	98.16	0.02388	− 0.75623 (ns)	0.2237
	G2 (n = 10)	650	1950	99.25	98.97	0.01589	0.63196 (ns)	0.1048
	G3 (n = 15)	642–650	1926–1950	96.48	97.67	0.02627	− 0.15383 (ns)	0.1962
ORF1/2 (RdRP)	all (n = 47)	1053–1070	3159–3210	91.45	94.17	0.07324	0.96401 (ns)	0.3757
	G1 (n = 22)	1053–1070	3159–3210	95.02	98.35	0.02163	− 0.67551 (ns)	0.3594
	G2 (n = 10)	1070	3210	97.64	98.69	0.01948	0.77878 (ns)	0.0884
	G3 (n = 15)	1062–1070	3186–3210	95.02	98.14	0.02109	− 0.29946 (ns)	0.2980
ORF3 (CP)	all (n = 47)	196	588	98.77	98.43	0.02242	− 0.14750 (ns)	0.2523
	G1 (n = 22)	196	588	99.72	99.71	0.0054	− 1.63275 (ns)	0.2138
	G2 (n = 10)	196	588	99.85	99.63	0.00606	− 0.34066 (ns)	0.1165
	G3 (n = 15)	196	588	99.29	99.53	0.0078	− 0.95978 (ns)	0.1567
ORF4 (MP)	all (n = 47)	150	450	98.85	99.03	0.01463	− 0.65870 (ns)	0.2244
	G1 (n = 22)	150	450	99.61	99.74	0.00441	− 1.71042 (ns)	0.3124
	G2 (n = 10)	150	450	99.76	99.89	0.00209	− 1.00300 (ns)	1.3254
	G3 (n = 15)	150	450	99.11	99.58	0.00668	− 0.85026 (ns)	0.6926
ORF3/5 (RTD)	all (n = 47)	675–676	2025–2028	94.29	94.30	0.07779	0.76678 (ns)	0.2235
	G1 (n = 22)	676	2028	98.05	98.17	0.02737	− 0.48646 (ns)	0.1782
	G2 (n = 10)	676	2028	99.20	98.95	0.0174	− 0.31252 (ns)	0.1381
	G3 (n = 15)	674–675	2022–2025	97.79	98.0	0.02983	− 0.40820 (ns)	0.1736

aa, amino acid; nt, nucleotide; π, nucleotide diversity; ns, not significant (*P* > 0.05); nonsynonymous/synonymous ratio (dN/dS) < 1 (negative or purifying selection), dN/dS = 1 (neutral evolution), and dN/dS > 1 (positive selection).

### Neutrality tests and selection pressure analysis

In neutrality tests, Tajima’s D was used to assess the deviation from neutrality for all mutations among group-specific SCYLV gene sequences. The values of Tajima’s D were negative for *CP* and *MP* gene sequences in the overall population and in each phylogrouping subpopulation, indicating that purifying selection is likely acting on SCYLV populations as a result of these populations increasing (Table 2). However, Tajima’s D values were positive for P0, P1, RdRP, and RTD in the overall SCYLV population and for P1 and RdRP in the phylogroup-G2 subpopulation, indicating these populations were stable. However, all Tajima’s D statistics were not statistically significant (*P* > 0.05) in any populations, suggesting that all populations appear to be at demographic equilibrium.

To evaluate selection pressure on each SCYLV coding region, ratios of non-synonymous (dN) over synonymous (dS) mutation rates were calculated. Negative or purifying selection (dN/dS < 1) enhances the speed of elimination of deleterious mutations in genes and shapes a stable population genetic structure, whereas positive selection (dN/dS > 1) plays an important role in virus adaptability to environmental changes and new hosts. Except for MP, the dN/dS ratio was < 1 for all SCYLV coding regions in most of subpopulations, indicating that purifying selection was a main factor restricting variability in the SCYLV population (Table 2). However, the purifying selective pressure was not distributed uniformly across the SCYLV genome in a specific subpopulation. For example, for genes in the phylogroup-G2 subpopulation, the strongest purifying selection appeared in RdRP (dN/dS = 0.0884), but positive selection was found in MP (dN/dS = 1.3254). The dN/dS > 1 for MP in the phylogroup-G2 subpopulation suggests positive selection is acting on this gene in this subpopulation.

### Population differentiation based on geographical origins

To shed light on the genetic relationship between different geographical populations, three statistics (Ks*, Z*, and Snn) were used to evaluate population genetic differentiation. Overall, significantly high values for the three statistics were obtained in all subpopulations based on each gene, indicating that the isolates between geographical populations had a very high genetic differentiation (Table 3). Furthermore, infrequent gene flow between Africa and the two subpopulations (Asia and America) were found, supported by an allele frequency across populations (Fst) > 0.33 (except for Fst > 0.20 in CP and MP) and a migration rate (Nm) < 1 for each gene (Table 3). However, frequent gene flow between Asian and American subpopulations was observed, as evidenced by an Fst < 0.1 (from 0.01581 in RdRP to 0.08389 in MP) and Nm > 1 (from 5.46 in MP and 31.13 in RdRP) for individual genes. Based on these statistics, geographical isolation likely played an important role in the SCYLV subpopulation structure of Africa. On the other hand, the five aforementioned parameters were also calculated for pairwise comparison between the SCYLV phylogrouping subpopulations. These results demonstrated significantly high values of Ks*, Z*, and Snn statistics for each gene between SCYLV phylogrouping subpopulations (except for MP gene in the comparison of G1 versus G2 subpopulations), indicating that the isolates between most phylogrouping subpopulations had a very high genetic differentiation and infrequent gene flow (Fst > 0.33 and Nm < 1; Table S3).

**Table 3:** Gene flow and genetic differentiation among sugarcane yellow leaf virus (SCYLV) subpopulations based on geographic origins.

Genomic region	Comparisons	Ks* (*P* value)	Z* (*P* value)	Snn (*P* value)	Fst	Nm
ORFs 0–5	Asia (n = 23) versus Africa (n = 10)	5.23017 (0.0000***)	6.24901 (0.0000***)	1.00000 (0.0000***)	0.45497	0.60
	Asia (n = 23) versus America (n = 14)	5.54500 (0.0030**)	6.77899 (0.0000***)	0.94595 (0.0000***)	0.05951	7.90
	Africa (n = 10) versus America (n = 14)	5.03290 (0.0000***)	5.48287 (0.0000***)	1.00000 (0.0000***)	0.40116	0.75
ORF0 (P0)	Asia (n = 23) versus Africa (n = 10)	3.52869 (0.0000***)	6.18396 (0.0000***)	1.00000 (0.0000***)	0.43323	0.65
	Asia (n = 23) versus America (n = 14)	3.94367 (0.0130*)	6.79418 (0.0000**)	1.00000 (0.0000***)	0.02270	21.52
	Africa (n = 10) versus America (n = 14)	3.07647 (0.0000***)	5.48663 (0.0000***)	1.00000 (0.0000***)	0.34610	0.94
ORF1 (P1)	Asia (n = 23) versus Africa (n = 10)	4.25633(0.0000***)	6.18533 (0.0000***)	1.00000 (0.0000***)	0.47999	0.54
	Asia (n = 23) versus America (n = 14)	4.57621 (0.0080**)	6.79286 (0.0000***)	1.00000 (0.0000***)	0.05817	8.10
	Africa (n = 10) versus America (n = 14)	3.94750 (0.0000***)	5.46695 (0.0000***^)^	1.00000 (0.0000***)	0.42363	0.68
ORF1-2 (RdRP)	Asia (n = 23) versus Africa (n = 10)	4.55296 (0.0000***)	5.96501 (0.0000***)	1.00000 (0.0000***)	0.42196	0.68
	Asia (n = 23) versus America (n = 14)	4.83504 (0.0140*)	6.56737 (0.0030**)	0.93939 (0.0000***)	0.01581	31.13
	Africa (n = 10) versus America (n = 14)	4.42390 (0.0000***)	5.50327 (0.0000***)	1.00000 (0.0000***)	0.41083	0.72
ORF3 (CP)	Asia (n = 23) versus Africa (n = 10)	1.99034 (0.0000***)	6.42184 (0.0000***)	0.94805 (0.0000***)	0.38354	0.80
	Asia (n = 23) versus America (n = 14)	2.22958 (0.0120*)	6.83625 (0.0000***)	0.85621 (0.0000***)	0.06444	7.26
	Africa (n = 10) versus America (n = 14)	1.84867 (0.0000***)	5.74703 (0.0000***)	0.92024 (0.0000***)	0.27405	1.32
ORF3-5 (RTD)	Asia (n = 23) versus Africa (n = 10)	4.22064 (0.0000***)	6.21377 (0.0000***)	1.00000 (0.0000***)	0.46086	0.58
	Asia (n = 23) versus America (n = 14)	4.59517 (0.0000***)	6.78249 (0.0000***)	0.95045 (0.0000***)	0.06138	7.65
	Africa (n = 10) versus America (n = 14)	4.06444 (0.0000***)	5.48591 (0.0000***)	1.00000 (0.0000***)	0.40357	0.74
ORF4 (MP)	Asia (n = 23) versus Africa (n = 10)	1.41525 (0.0000***)	6.44432 (0.0000***)	0.81567 (0.0000***)	0.40385	0.74
	Asia (n = 23) versus America (n = 14)	1.76195 (0.0130*)	6.80554 (0.0060**)	0.79410 (0.0000***)	0.08389	5.46
	Africa (n = 10) versus America (n = 14)	1.21246 (0.0000***)	5.86777 (0.0000***)	0.80136 (0.0000***)	0.20976	1.88

Symbols are showed for three permutation statistical tests of Ks*, Z*, and Snn at different statistical significance levels: *0.01 < *P* ≤ 0.05; **0.001 < *P* ≤ 0.01; ***P ≤ 0.001.

## Discussion

YLD is a very common viral disease, and at least six SCYLV genotypes (BRA, CHN1, CHN2, CHN3, CUB, and PER or HAW) occurr in China^28,30,31,35^. Previous studies showed that BRA genotype is most prevalent in Chinese sugarcane planting areas, which is also highly distributed around the word, but other genotypes just occurred in limited regions^28,30,31^. The REU and CUB genotypes from Réunion Island and Cuba, respectively, were proposed by Abu et al.^38^ based on the geographical origins where they were first determined. The SCYLV isolate (CHN-GD-WY20) of the CUB genotype was also present in Guangdong Province, China, but only one isolate is available^31^. However, information is limited about SCYLV genome sequences of REU and CUB genotypes to date. In the present study, two genomic sequences of REU genotype and four genomic sequences of CUB genotype were obtained which enrich the genomic information available on the two SCYLV genotypes.

Apart from SCYLV genotype CUB occurring in China, this genotype is also distributed in India^36^ and Mauritius^37^. It is likely that this virus was transmitted by germplasm exchanges among the countries through vegetative propagation cuttings^31,36,38^. Furthermore, it is no surprise that the CUB genotype was present in both provinces of China because the sugarcane variety ‘Guangdong Huangpi’ (a chewing cane) was introduced from Guangdong Province where the CUB genotype exists to Zhejiang Province in 2010. Notably, FL180 (MH058009) along with chn1 (GU327735) were clustered in SCYLV genotype CHN1 in the present study, similar to Filloux et al.^39^, because the two isolates were clustered together in a branch based on each protein. The reason for the close relationship between the two isolates is likely that isolate chn1 hosted in sugarcane cultivar CP93-1309 was developed in Florida, USA, and then exported to China^31^, while FL180 (MH058009) hosted in cultivar CP00-1101 also originated from Florida^39^. Three isolates Sorg1_1 (KY960995), Sorg2_2 (KY960996), and Sorg3_3 (KY960997) hosted in grain sorghum in Florida were not identified as a specific genotype^27,39^, but they were recently proposed as FLA1, LFA2, and FLA3 genotypes, respectively, based on geographical origin^29^. Therefore, the current results suggest isolate FL84 (MH058007) from sugarcane together with two isolates (Sorg1_1 and Sorg2_2) clustered into a branch be assigned the FLA1/2 genotype, while Sorg3_3 is assigned a distinct FLA3 genotype^29^. More genomic sequences of SCYLV are needed to further discriminate between FLA1 and FLA2 genotypes.

Recombination is an important driving force in the evolution of poleroviruses, which contributes to genetic diversity in the virus population^17,40^. Numerous observations have indicated that RNA recombination occurs frequently in SCYLV based on nearly complete genome sequences^27,28,32^. Similar to observations by ElSayed et al. ^27^, the current data also demonstrated that recombination events among worldwide SCYLV isolates occurred in inter-phylogroups between geographical locations and crops (sugarcane and grain sorghum). These cases of genomic exchange are likely due to human exchange of sugarcane materials resulting in transmission through traditional geographical barriers between plant viruses^28^. Recombination events may create a new virus or strain, expand host range, or modify vector specificity^40^. The present findings further showed that most recombination hotspots were distributed in the SCYLV RTD protein. Notably, RTD protein encoded by poleroviruses is essential for aphid transmission and virus movement in plants^17^. Therefore, recombination hotspots of SCYLV isolates frequently present in RTD protein may help this virus accomplish jumping from sugarcane to grain sorghum as a host.

Other common sequence variation mechanisms, such as InDel and codon mutations, are also important driving forces in the evolution of plant viruses, including poleroviruses^41^. Sequence variation in the SCYLV genome may affect the degree of infection capacity, virulence, transmission, and symptoms^28,42^. The present data revealed the highest sequence divergence in P0 encoded by ORF0, followed by P1 encoded by ORF1. The P0 protein of poleroviruses, including SCYLV encoding a suppressor of RNA silencing, suppresses either local or both local and systemic gene silencing in host plants^17,43^. This SCYLV P0 protein affects disease symptoms^43^, and various P0 proteins from different viral genotypes diverge on suppression of host RNA silencing activity^44^, suggesting the performance of high sequence variability in SCYLV P0 probably contributes to different biological functions for this protein. On the other hand, P1 contains two putative domains, viral protein genome-linked (VPg) and a protease that releases VPg, when P1 is expressed by itself and not fused with P2^17^. Observations from past studies suggest that deletions in ORF1 are associated with the high proliferation rate of the virus in susceptible plants^26,30^. Recombination together with mutation is an important factor in RNA viruses having a broad host range or using several vector species for transmission^28,45^.

The current data showed that geographic isolation contributed to shaping the genetic structure of SCYLV populations, particularly between Africa and two other regions (Asia and America), as evidenced by low-level gene flow and significant genetic differentiation between SCYLV geographic populations. This geographical distribution and phylogeny were consistent with our previous study based on P0 and P1 proteins^46^. Similarly, geographical isolation is responsible for the population structures of maize yellow mosaic virus, which infects sugarcane and maize (*Zea mays*) and is a novel species in the genus *Polerovirus*^41^. Additionally, the present findings showed that the six proteins encoded by SCYLV isolates from individual geographic populations were negative for purification selection, suggesting that this evolutionary driving force may enhance the stability of SCYLV population genetic structure. However, the *MP* gene of SCYLV from the African population is under positive selection, suggesting it may serve an important role in SCYLV adaptability to locational environment conditions. Other similar investigations have indicated that *MP* genes from viruses in the family *Luteoviridae* underwent positive selection pressure, but other genes were under purifying selection^41,47^.

Overall, the present study examined genetic variability and molecular evolution among SCYLV populations using comparative genomics, improving the current understanding of the genotypic variability of SCYLV populations throughout the world and enriching the taxonomic status of SCYLV isolates. High genetic diversity occurred among these SCYLV isolates. Recombination and InDels contributed to genetic variation during the evolutionary history of SCYLV. Geographical isolation (particularly in Africa) and purifying selection (except for *MP* gene) are important evolutionary driving forces shaping the SCYLV subpopulation. YLD is commonly managed by resistant cultivars and disease-free seedcane. Quarantine is also important to prevent exotic strains of SCYLV from entering counties where YLD is not present. The present results provide a research basis for transgenic breeding to generate new disease-resistant cultivars, as well as more valuable information for developing efficient molecular detection approaches of the virus during the quarantine stage. The present work also points out that to prevent the spread of YLD, healthy cutting is essential for transferring sugarcane material across borders.

## Methods

### Leaf sample collection and RNA extraction

Two symptomatic leaves were collected from a local sugarcane clone in Réunion Island in November 2015 while four asymptomatic leaves were collected from a chewing cane cultivar ‘Guangdong Huangpi’ in Wenling, Zhejiang Province, China, in June 2019. Different leaves were collected from different sugarcane plants from the same fields. The collection of leaf samples has been permitted by eRcane, Réunion Island, France. Total RNA was extracted from leaf samples using TRIzol Reagent (Invitrogen, Carlsbad, CA, USA) according to the manufacturer’s protocol. The final concentration (100 ng/µL) of total RNA was used for SCYLV detection and viral genome cloning.

### SCYLV detection by RT-PCR

The SCYLV-specific primers YLS111 and YLS462 designed by Dr. Mike Irey (US Sugar Corporation, Florida, USA) were used for SCYLV detection in the collected leaf samples with RT-PCR^30^. RT reactions were performed in a 10 µL total volume containing total RNA using a PrimeScript RT Reagent Kit (TaKaRa, Dalian, China) according to the manufacturer’s instructions. PCR reactions were conducted using Ex-Taq PCR Master Mix (TaKaRa Biotech, Dalian, China) and PCR cycling parameters were as follows: an initial denaturation at 94 °C for 2 min, 35 cycles of 94 °C for 30 s, 56 °C for 45 s, and 72 °C for 2 min, with a final 72 °C extension for 10 min.

### Genome fragment cloning and assembly

In order to clone and assemble the complete genome sequences of SCYLV isolates, RT-PCR was carried out using five sets of primer pairs designed by Lin et al. ^28^ for Réunion Island samples and three sets of primer pairs designed herein for Chinese samples (Table S4). RT reactions were conducted with the same protocol used in SCYLV detection. PCR reactions were performed using Ex-Taq PCR Master Mix (TaKaRa Biotech) following the protocol described by Lin et al.^28^, with minor modification, such as an annealing temperature based on the Tm (°C) of each primer pair (Table S4). PCR-amplified genomic fragments were ligated into a pMD19-T vector (TaKaRa Biotech). Three independent clones for each amplicon were sequenced in both directions by Sangon Biotech Co, Ltd, (Shanghai, China). To remove errors caused by in vitro PCR, a consensus sequence was generated when the three independent clones per isolate showed ≥ 99% identity among three contigs. The full-length genome sequences of six SCYLV isolates (Réunion Island = 2, China = 4) were assembled from several overlapping fragments using DNAMAN 8 (Lynnon Biosoft, San Ramon, CA, USA).

### Sequence alignment and phylogenetic analysis

In total, 50 complete genome sequences of SCYLV isolates (present study = 6, GenBank = 44) were used for sequence analysis. These isolates originated from different areas (Asia = 18, Africa = 10, and America = 22; Table S1). Six additional datasets of nucleotide and amino acid sequences from each gene and protein were also analyzed. All datasets were aligned using the ClustalW algorithm in MEGA7^48^. Phylogenetic analysis was carried out using IQ-TREE version 1.6.12 software to construct a ML tree with TIM2e + R5 model and 1000 bootstrap replications^49^. The genome sequence of potato leafroll virus (GenBank accession no. NC_001747) was used as an outgroup. Meanwhile, pairwise sequence identity analysis was conducted by BioEdit version 7.1.9^50^ and pairwise identity matrix inferred using SDT v1.2^51^.

### Test for recombination

Recombination analysis was tested among the 50 complete genome sequences (ORFs 0–5) using RDR4 v 4.69^52^. Seven different recombination detection algorithms (RDP, GENECONV, Chimaera, MaxChi, Bootscane, SISCAN, and 3Seq) implemented in RDP4 were used with default parameters. Recombination events that were detected by at least four of the seven detection algorithms with significant statistical support and Bonferroni-corrected *P* value cutoff of 0.05 were considered acceptable^28,32^. Furthermore, to verify the authenticity of the recombination events, Simplot 3.5.1 software was used to test putative recombination events according to the consistency between the potential recombination isolate and its major and minor parents^53^.

### Calculation of population genetic parameters

Three SCYLV isolates (Sorg1_1, Sorg2_2, and Sorg3_3) from grain sorghum were excluded from the below-described analysis because their complete genes and proteins were not available in the GenBank database. InDel analysis was manually calculated based on the aligned sequences of SCYLV. Nucleotide diversity (π) between SCYLV groups was estimated according to Nei^54^. Neutrality testing of Tajima’s D^55^ was performed in DnaSP version 5.10.01 software^56^. To estimate selection pressure on each SCYLV coding region, the dN/dS ratio was calculated using the HYPhy package^57^. To assess genetic differentiation between SCYLV populations from different geographical origins (Asia, Africa, and America), three permutation statistical tests (Ks*, Z*, and Snn) were performed with 1000 replicates^58,59^. If the test statistics were strongly supported by *P* values < 0.05, the null hypothesis of no genetic differentiation was rejected. Meanwhile, the standardized variance of Fst (Subpopulation fixation index) and Nm (Number of migrants) were used for the degree of gene flow between SCYLV populations. If |Fst|> 0.33 or |Nm|< 1, infrequent gene flow is accepted to have occurred. If |Fst|< 0.33 or |Nm|> 1, frequent gene flow is considerable to have occurred^41^. All of the population genetic parameters were performed by DnaSP software.

## Supplementary Information

Supplementary Information 1.

Supplementary Figure S1.

Supplementary Table S1.

Supplementary Table S2.

Supplementary Table S3.

Supplementary Table S4.

## Data Availability

The data that support the findings of this manuscript are available from the corresponding author upon reasonable request.
