# Supplementary material for: Comparative genomics reveals insights into genetic variability and molecular evolution among sugarcane yellow leaf virus populations
Source: Sci Rep. 2021 Mar 30;11:7149. doi: 10.1038/s41598-021-86472-z (PMC8009895; doi:10.1038/s41598-021-86472-z)
Supplement: Supplementary file 1 — Supplementary Information 1. [file 41598_2021_86472_MOESM1_ESM.docx]

**Supporting information**

**Comparative genomics reveals insights into genetic variability and molecular evolution among sugarcane yellow leaf virus populations**

Jia-Ju Lu^1,†^, Er-Qi He^1,2,†^, Wen-Qing Bao^2^, Jian-Sheng Chen^2^, Sheng-Ren Sun^2^, San-Ji Gao^2,*^

^1^Guizhou Institute of Subtropical Crops, Guizhou Academy of Agricultural Sciences, Xingyi 562400, Guizhou, China;

^2^National Engineering Research Center for Sugarcane, Fujian Agriculture and Forestry University, Fuzhou, Fujian 350002, China

^†^These authors have contributed equally to this work.

^*^Correspondence: gaosanji@fafu.edu.cn

**Supplementary data**

**Supplementary Tables**

**Table S1.** Sources of sugarcane yellow leaf virus (SCYLV) sequences used in the present study.

**Table S2.** Sequence identity (%) within and between sugarcane yellow leaf virus (SCYLV) phylogroups based on the nucleotide (lower-left) and amino acid sequence (upper-right).

**Table S3.** Gene flow and genetic differentiation among sugarcane yellow leaf virus (SCYLV) subpopulations based on phylogroups.

**Table S4.** The primers used for cloning sugarcane yellow leaf virus (SCYLV) genomes.

**Supplementary Figure**

**Figure S1.** Schematic diagram of recombination events among 50 sugarcane yellow leaf virus (SCYLV) isolates identified by Simplot software. Putative recombinant isolates were: (a) PI 157033 (IND2 × BRA-YL1); (b) Sorg1_1 (FL86 × FL84); (c) MU-AB193 (CHN-GD-ZJ4 × MU-AB197); (d) Sorg3_3 (SCYLV × SCYLV-ZJWL003); (e) MU-SC1233 (CHN-YN-KY2 × MU-AB193); (f) MU-AB193 (PI 157033 × REU-YL2); (g) MU-SC1233 (CHN-GD-ZJ15 × MU-AB197); (h) GZ-GZ18 (CHN-GD-WY19 × FL86); (i) Sorg2_2 (FL86 × IND2); (j) Sorg3_3 (SCYLV × FL180); (k) Sorg1_1 (PI 157033 × Sorg3_3); (l) IND2 (FL180 × CBLK97154). Recombination regions are marked with red boxes.
