# Supplementary material for: Comparative genomics reveals insights into genetic variability and molecular evolution among sugarcane yellow leaf virus populations
Source: Sci Rep. 2021 Mar 30;11:7149. doi: 10.1038/s41598-021-86472-z (PMC8009895; doi:10.1038/s41598-021-86472-z)
Supplement: Supplementary file 2 — Supplementary Figure S1. [file 41598_2021_86472_MOESM2_ESM.pdf]

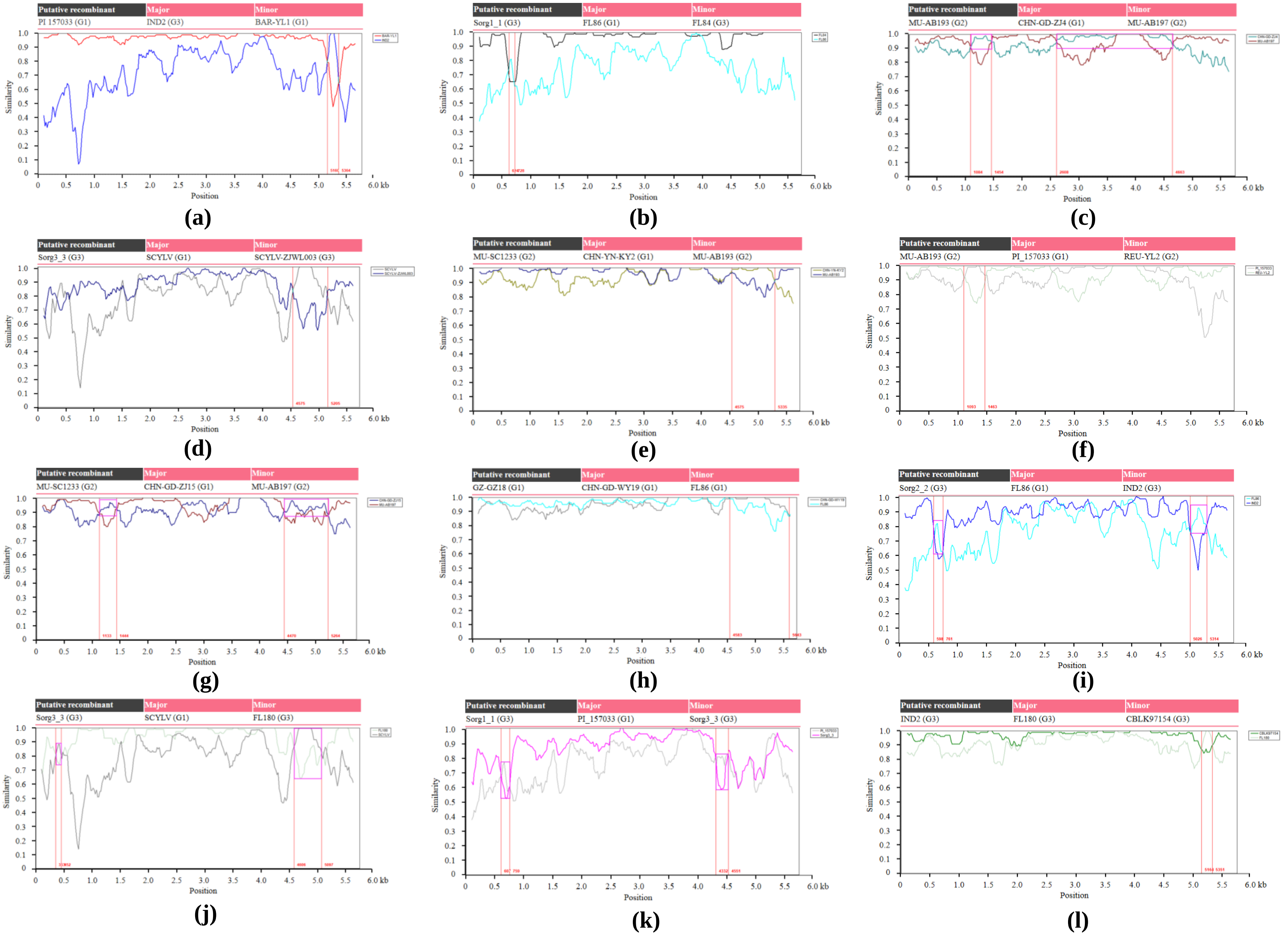

Figure S1. Schematic diagram of recombination events among 50 sugarcane yellow leaf virus (SCYLV) isolates identified by Simplot software. Putative recombinant isolates were: (a) PI 157033 (IND2 × BRA-YL1); (b) Sorg1_1 (FL86 × FL84); (c) MU-AB193 (CHN-GD-ZJ4 × MU-AB197); (d) Sorg3_3 (SCYLV × SCYLV-ZJWL003); (e) MU-SC1233 (CHN-YN-KY2 × MU-AB193); (f) MU-AB193 (PI 157033 × REU-YL2); (g) MU-SC1233 (CHN-GD-ZJ15 × MU-AB197); (h) GZ-GZ18 (CHN-GD-WY19 × FL86); (i) Sorg2_2 (FL86 × IND2); (j) Sorg3_3 (SCYLV × FL180); (k) Sorg1_1 (PI 157033 × Sorg3_3); (l) IND2 (FL180 ×CBLK97154). Recombination regions are marked with red boxes.
