# Supplementary material for: Comparative genomics reveals insights into genetic variability and molecular evolution among sugarcane yellow leaf virus populations
Source: Sci Rep. 2021 Mar 30;11:7149. doi: 10.1038/s41598-021-86472-z (PMC8009895; doi:10.1038/s41598-021-86472-z)
Supplement: Supplementary file 3 — Supplementary Table S1. [file 41598_2021_86472_MOESM3_ESM.pdf]

Table S1. Sources of sugarcane yellow leaf virus (SCYLV) sequences used in the present study.

Genotype/Grouping[a]	Isolate name	Host palnt	Genome size (bp)	Location/Country	Region	GenBank accession no.	Reference
BRA/G1	SCYLV-A	CP65-357	5899	Florida, USA	North America	AF157029	Moonan et al., 2000
BRA/G1	BRA-YL1	SP71-6163	5612	Brazil	South America	AM072750	Abu Ahmad et al., 2006
BRA/G1	CHN-YL1	CGT63-167	5612	China	Asia	AM072751	Abu Ahmad et al., 2006
BRA/G1	CHN-HN1	ROC22	5837	Hainan, China	Asia	HQ342888	Wang et al., 2012
BRA/G1	CHN-GD-ZJ4	-	5718	Guangdong, China	Asia	HQ245319	Wang et al., 2012
BRA/G1	CHN-GD-ZJ17	-	5716	Guangdong, China	Asia	HQ245321	Wang et al., 2012
BRA/G1	CHN-YN-KY2	Yunrui99-601	5716	Yunnan, China	Asia	HQ245322	Wang et al., 2012
BRA/G1	CHN-FJ1	FN96-0907	5879	Fujian, China	Asia	GU190159	Gao et al., 2012
BRA/G1	FL86	CL91-4814	5796	USA	South America	MH058008	Filloux et al., 2018
BRA/G1	SCYLV	-	5899	India	Asia	AY236971	Direct submission
BRA/G1	PI 157033	*Sorghum bicolor*	5891	Florida, USA	North America	MN097766	Direct submission
CHN3/G1	GZ-GZ18	Ganzhe18	5880	Guizhou, China	Asia	KF477092	Lin et al., 2014
CHN3/G1	HN-CP502	CP49-50	5880	Hainan, China	Asia	KF477093	Lin et al., 2014
HAW/G1	CP65-357	CP65-357	5895	Florida, USA	North America	AJ249447	Smith et al., 2000
HAW/G1	Haw87-4319	H87-4319	5770	Hawaii, USA	North America	GU570006	ElSayed et al., 2011
HAW/G1	Haw87-4094	H87-4094	5821	Hawaii, USA	North America	GU570007	ElSayed et al., 2011
HAW/G1	Haw73-6110	H73-6110	5773	Hawaii, USA	North America	GU570008	ElSayed et al., 2011
PER/G1	PER-YL1a	H50-7209	5612	Peru	South America	AM072752	Abu Ahmad et al., 2006
PER/G1	PER-YL1b	H50-7209	5612	Peru	South America	AM072753	Abu Ahmad et al., 2006
PER/G1	CHN-GD-JM2	-	5716	Guangdong, China	Asia	HQ245316	Wang et al., 2012
PER/G1	CHN-GD-WY19	-	5716	Guangdong, China	Asia	HQ245317	Wang et al., 2012

PER/G1	CHN-GD-ZJ15	-	5716	Guangdong, China	Asia	HQ245320	Wang et al., 2012
REU/G2	REU-YL11	*Saccharum* spp. hybrid	5881	Saint-Gilles, Réunion Island	Africa	KY052165	This study
REU/G2	REU-YL15	*Saccharum* spp. hybrid	5881	Saint-Gilles, Réunion Island	Africa	KY052166	This study
REU/G2	REU-YL1a	R570	5612	La Mare, Reunion Island	Africa	AM072754	Abu Ahmad et al., 2006
REU/G2	REU-YL1b	R570	5612	La Mare, Reunion Island	Africa	AM072755	Abu Ahmad et al., 2006
REU/G2	REU-YL2	R490	5612	La Mare, Reunion Island	Africa	AM072756	Abu Ahmad et al., 2006
REU/G2	MU-AB193	R570	5854	Mauritius	Africa	MF197922	Direct submission
REU/G2	MU-AB194	R570	5854	Mauritius	Africa	MF197923	Direct submission
REU/G2	MU-SC1233	R579	5854	Mauritius	Africa	MF197925	Direct submission
REU/G2	MU-AB110	M2024/88	5854	Mauritius	Africa	MF197921	Direct submission
REU/G2	MU-AB197	R570	5854	Mauritius	Africa	MF197924	Direct submission
Chn1/G3	SCYLV-chn1	CP93-1309	5803	Guangdong, China	Asia	GU327735	Wang and Zhou, 2010
CUB/G3	CHN-GD-WY20	-	5744	Guangdong, China	Asia	HQ245318	Wang et al., 2012
CUB/G3	COL-YL1a	CC87-505	5811	Colombia	South America	MF622078	Direct submission
CUB/G3	CUB-YL1a	C132-81	5810	Cuba	North America	MF622079	Direct submission
CUB/G3	CBLK97154	CBLk 97154	5805	Lucknow, India	Asia	KF680098	Direct submission
CUB/G3	ZJWL002	Guangdong Huangpi	5775	Zhejiang,China	Asia	MW439312	This study
CUB/G3	ZJWL003	Guangdong Huangpi	5776	Zhejiang,China	Asia	MW439313	This study
CUB/G3	ZJWL007	Guangdong Huangpi	5776	Zhejiang,China	Asia	MW446950	This study
CUB/G3	ZJWL012	Guangdong Huangpi	5776	Zhejiang,China	Asia	MW446951	This study
IND/G3	IND1	B 38192	5875	Coimbatore, India	Asia	JF925152	Chinnaraja et al., 2013
IND/G3	IND2	Co 86010	5875	Coimbatore, India	Asia	JF925153	Chinnaraja et al., 2013

IND/G3	IND3	CoV 92102	5870	Coimbatore, India	Asia	JF925154	Chinnaraja et al., 2013
IND/G3	IND4	CoC 85061	5878	Coimbatore, India	Asia	JF925155	Chinnaraja et al., 2013
CHN1/G3	FL180	CP00-1101	5789	USA	South America	MH058009	Filloux et al., 2018
FLA1 or 2/G3	FL84	IJ76-478	5776	USA	South America	MH058007	Filloux et al., 2018
FLA1 or 2/G3	Sorg1-1	*Sorghum bicolor* cv. Keller	5748	USA	North America	KT960995	ElSayed et al., 2018
FLA1 or 2/G3	Sorg2_2	*Sorghum bicolor* cv. Dale	5840	USA	North America	KT960996	ElSayed et al., 2018
FLA3/G3	Sorg3_3	*Sorghum bicolor* cv. Top76-6	5821	USA	North America	KT960997	ElSayed et al., 2018

[a]The acronyms of each SCYLV genotype based on their originally geographical origins: BRA (Brazil), HAW (Hawaii), PER (Peru), CHN (China), REU (Reunion), FLA (Florida), IND (India), and CUB (Cuba).
