# Supplementary material for: Comparative genomics reveals insights into genetic variability and molecular evolution among sugarcane yellow leaf virus populations
Source: Sci Rep. 2021 Mar 30;11:7149. doi: 10.1038/s41598-021-86472-z (PMC8009895; doi:10.1038/s41598-021-86472-z)
Supplement: Supplementary file 4 — Supplementary Table S2. [file 41598_2021_86472_MOESM4_ESM.pdf]

Table S2. Sequence identity (%) within and between sugarcane yellow leaf virus (SCYLV) phylogroups based on the nucleotide (lower-left) and amino acid sequence (upper-right).

Genomic region	Subpopulation	G1(n = 22)	G2(n = 10)	G3(n = 18/n = 15) [a]
ORF1-5	G1(n=22)	95.1-99.9		
	G2(n=10)	92.7-96.7	97.0-99.7	
	G3(n=18)	83.7-87.6	84.4-87.3	88.8-99.4
ORF0	G1(n=22)	95.7-99.8(93.3-100)	93.3-98.0	68.3-73.4
	G2(n=10)	94.7-97	97.4-99.8(96.4-100)	70.7-73.8
	G3(n=15)	76.1-78.5	77.3-79.2	94.5-99.7(94.9-100)
ORF1	G1(n=22)	92.2-99.8(92.1-100)	91.3-96.0	72.1-81.2
	G2(n=10)	90.8-96.3	93.0-99.8(97.5-99.8)	73.3-80.4
	G3(n=15)	79.9-83.9	81.7-84.1	93.0-99.7(88.6-99.6)
ORF1-2	G1(n=22)	95.0-99.8(81.1-100)	81.5-97.0	70.1-87.1
	G2(n=10)	92.0-96.6	96.4-99.6(80.0-99.7)	79.3-87.3
	G3(n=15)	84.6-87.3	86.0-87.9	94.8-99.8(88.7-99.7)
ORF3	G1(n=22)	98.6-100(97.9-100)	98.4-100	93.3-100
	G2(n=10)	97.9-100	98.4-100(98.9-100)	93.8-98.9
	G3(n=15)	94.9-96.2	95.0-96.6	97.9-100(93.3-100)
ORF3-5	G1(n=22)	93.5-99.9(94.3-99.8)	86.6-98.8	80.1-98.6
	G2(n=10)	92.1-96.1	93.9-100(91.8-99.8)	84.3-88.6
	G3(n=15)	84.0-89.2	85.5-87.4	93.9-99.4(93.9-100)
ORF4	G1(n=22)	98.4-100(97.3-100)	97.3-100	95.3-98.0
	G2(n=10)	98.8-100	99.3-100(98.0-100)	95.3-98.0
	G3(n=15)	96.2-97.7	96.4-97.7	98.2-100(97.3-100)

[a] The 18 SCYLV sequences in phylogroup G3 were used for the analysis of nearly complete genome sequence (ORF1-5), while 15 sequences in G3 were used for the analysis of individual genes/proteins because these detailed ORFs are not available in three SCYLV isolates (Sorg1_1, Sorg2_2, and Sorg3_3) from grain sorghum.
