# Supplementary material for: Comparative genomics reveals insights into genetic variability and molecular evolution among sugarcane yellow leaf virus populations
Source: Sci Rep. 2021 Mar 30;11:7149. doi: 10.1038/s41598-021-86472-z (PMC8009895; doi:10.1038/s41598-021-86472-z)
Supplement: Supplementary file 5 — Supplementary Table S3. [file 41598_2021_86472_MOESM5_ESM.pdf]

Table S3. Gene flow and genetic differentiation among sugarcane yellow leaf virus (SCYLV) subpopulations based on phylogroups [a].

Genomic region	Comparisons	Ks*(*p*-value)	Z*(*p*-value)	Snn(p-value)	Fst	Nm
ORFs 0-5	G1 (n=22) vs. G2 (n=10)	4.63127(0.0000 [***])	5.98158(0.0000 [***])	1.00000(0.0000 [***])	0.5839	0.3563
	G1 (n=22) vs. G3 (n=15)	4.73841(0.0000 [***])	6.23193(0.0000 [***])	1.00000(0.0000 [***])	0.81401	0.1142
	G2 (n=10) vs. G3 (n=15)	4.59318(0.0000 [***])	5.41772(0.0000 [***])	1.00000(0.0000 [***])	0.83047	0.1021
ORF0 (P0)	G1 (n=22) vs. G2 (n=10)	2.67343(0.0000 [***])	5.90896(0.0000 [***])	1.00000(0.0000 [***])	0.53572	0.4333
	G1 (n=22) vs. G3 (n=15)	2.96167(0.0000 [***])	6.26669(0.0000 [***])	1.00000(0.0000 [***])	0.87724	0.0700
	G2 (n=10) vs. G3 (n=15)	2.71735(0.0000 [***])	5.35313(0.0000 [***])	1.00000(0.0000 [***])	0.90088	0.0550
ORF1 (P1)	G1 (n=22) vs. G2 (n=10)	3.53753(0.0000 [***])	5.94487(0.0000 [***])	1.00000(0.0000 [***])	0.63075	0.2927
	G1 (n=22) vs. G3 (n=15)	3.68511(0.0000 [***])	6.24086(0.0000 [***])	1.00000(0.0000 [***])	0.84471	0.0919
	G2 (n=10) vs. G3 (n=15)	3.5398(0.0000 [***])	5.39769(0.0000 [***)]	1.00000(0.0000 [***])	0.86843	0.0758
ORF1-2 (RdRP)	G1 (n=22) vs. G2 (n=10)	4.02186(0.0000 [***])	6.00093(0.0000 [***])	1.00000(0.0000 [***])	0.60851	0.3217
	G1 (n=22) vs. G3 (n=15)	4.04859(0.0000 [***])	6.2286(0.0000 [***])	1.00000(0.0000 [***])	0.83176	0.1011
	G2 (n=10) vs. G3 (n=15)	3.98212(0.0000 [***])	5.42773(0.0000 [***])	1.00000(0.0000 [***])	0.83332	0.1000
ORF3 (CP)	G1 (n=22) vs. G2 (n=10)	1.31512(0.0000 [***])	6.32354(0.0000 [***])	0.94035(0.0000 [***])	0.44556	0.6222
	G1 (n=22) vs. G3 (n=15)	1.40924(0.0000 [***])	6.29213(0.0000 [***])	1.00000(0.0000 [***])	0.84303	0.0931
	G2 (n=10) vs. G3 (n=15)	1.4872(0.0000 [***])	5.44395(0.0000 [***])	1.00000(0.0000 [***])	0.82529	0.1058
ORF3-5 (RTD)	G1 (n=22) vs. G2 (n=10)	3.6838(0.0000 [***])	5.98905(0.0000 [***])	1.00000(0.0000 [***])	0.55989	0.3930
	G1 (n=22) vs. G3 (n=15)	3.85729(0.0000 [***])	6.2359(0.0000 [***])	1.00000(0.0000 [***])	0.78179	0.1396
	G2 (n=10) vs. G3 (n=15)	3.65(0.0000 [***])	5.40513(0.0000 [***])	1.00000(0.0000 [***])	0.82055	0.1093
ORF4 (MP)	G1 (n=22) vs. G2 (n=10)	0.87396(0.0020 [**])	6.64713(0.0070 [**])	0.77108(0.0000 [***])	0.07416	6.2422
	G1 (n=22) vs. G3 (n=15)	1.12089(0.0000 [***])	6.31522(0.0000 [***])	1.00000(0.0000 [***])	0.79988	0.1251
	G2 (n=10) vs. G3 (n=15)	1.00274(0.0000 [***])	5.41224(0.0000 [***])	1.00000(0.0000 [***])	0.83798	0.0967

[a] Symbols are showed for three permutation statistical tests of Ks*, Z*, and Snn at different statistical significance levels: **, $0.001 < P < 0.01$; ***, $P < 0.001$.
