# Supplementary material for: Comparative genomics reveals insights into genetic variability and molecular evolution among sugarcane yellow leaf virus populations
Source: Sci Rep. 2021 Mar 30;11:7149. doi: 10.1038/s41598-021-86472-z (PMC8009895; doi:10.1038/s41598-021-86472-z)
Supplement: Supplementary file 6 — Supplementary Table S4. [file 41598_2021_86472_MOESM6_ESM.pdf]

Table S4. The primers used for cloning sugarcane yellow leaf virus (SCYLV) genomes [a].

Amplified fragment	Primer name	Sequence (5'→3')	Amplicon size (bp)	Position [a]	Tm (℃)	Targeted genotype	Reference
From 5' UTR to partial ORF 2	5UTR-F1	ACAAAATATATCGGGAGGGAAAC	967	36-1002	56	CHN3/REU	This study
	ORF0-R2	GCCACATCTTGAAAAACATCC		506-526			Lin et al, 2014
From partial ORF0 to ORF 1	ORF0-1BF	GGGAGGTTTCCAGGTGTTTGA	1514	139-159	60	CHN3/REU	Lin et al, 2014
	ORF0-1BR	GGTGACTTTGCCTCAACGACA		1632-1652			
From partial ORF 1 to ORF 2	ORF1-2CF	TGATTACGCCGAGGAGGTG	1387	1527-1545	62	CHN3/REU	Lin et al, 2014
	ORF1-2CR	TGTCGTCTTCGAGCATCCAG		2894-2913			
From partial ORF 2 to ORF 5	B FOR	GGATTGTGCGATCCGATTCG	1880	2562-2581	59	CHN3/REU	Abu Ahmad et al,2006
	B REV	CAGTTGCTCAATGCTCCACG		4422-4441			
Partial ORF5	104R.613R	ATATCTAGATGTGGGTCCGC	1300	4366-4385	52	CHN3/REU	Abu Ahmad et al,2006
	3'PRIME2	GGAGGAGGAAGATTTCGGTG		5646-5665			
From partial ORF5 to 3' UTR	ORF5 FOR	CTAACGCTTCACGTTCAGCC	588	5292-5311	62	CHN3/REU	Abu Ahmad et al,2006
	ORF5 REV	GCAGTGCCTCCCTGTATTCC		5860-5879			
Partial ORF3	YLS111	TCTCACTTTCACGGTTGACG	352	3826-3845	56	All	Abu Ahmad et al,2006
	YLS462	GTCTCCATTCCCTTTGTACAGC		4156-4177			
From 5' UTR to partial ORF 2	5UTR-F1	ACAAAATATATCGGGAGGGAAAC	1002	1-22	55	CUB	This study
	SCYLV-A-R	GCATCCTGTGTNCGATTG		985-1002			
From partial ORF 1 to ORF 4	SCYLV-B-F	TACTTGAGGCGGAGGTTGTTC	3643	847-869	57	CUB	This study
	SCYLV-B-R	ATCGTGCCATACCTTATCTCCTC		4467-4489			
From partial ORF 3 to 3' UTR	SCYLV-E-F	ACGGAAGCCTAGCAGGACAAT	1604	4176-4196	58	CUB	This study
	SCYLV-E-R	GAAACTGGAGAACTCCGCAAAGTT		5758-5781			

[a] Primer position was referred from complete the genome nucleotide sequence of isolate SCYLV-A (GenBank accession no. AF157029).
